# Impact of pelvic floor muscle training on sexual function in women affected by stress urinary incontinence

**DOI:** 10.1093/sexmed/qfae040

**Published:** 2024-06-20

**Authors:** Hui-Hsuan Lau, Tsung-Hsien Su, Jiun-Chyi Hwang

**Affiliations:** Department of Medicine, Mackay Medical College, Sanzhi District, New Taipei City 252, Taiwan; Division of Urogynecology, Department of Obstetrics and Gynecology, Mackay Memorial Hospital, Zhongshan District, Taipei City 104, Taiwan; Mackay Medicine, Nursing and Management College, Beitou District, Taipei City 112, Taiwan; Department of Medicine, Mackay Medical College, Sanzhi District, New Taipei City 252, Taiwan; Division of Urogynecology, Department of Obstetrics and Gynecology, Mackay Memorial Hospital, Zhongshan District, Taipei City 104, Taiwan; Mackay Medicine, Nursing and Management College, Beitou District, Taipei City 112, Taiwan; Department of Medicine, Mackay Medical College, Sanzhi District, New Taipei City 252, Taiwan; Division of Urogynecology, Department of Obstetrics and Gynecology, Mackay Memorial Hospital, Zhongshan District, Taipei City 104, Taiwan

**Keywords:** pelvic floor muscle training, quality of life, stress urinary incontinence, sexual function

## Abstract

**Background:**

Pelvic floor muscle training can effectively improve pelvic floor muscle strength and activities; however, its impact on sexual function in women with stress urinary incontinence remains unclear.

**Aim:**

The study sought to investigate the impact of pelvic floor muscle training on pelvic floor muscle and sexual function in women with stress urinary incontinence.

**Methods:**

This was a retrospective observational study involving women who visited a urogynecologic clinic at a tertiary medical center. Patients with stress urinary incontinence without pelvic organ prolapse underwent pelvic floor muscle training programs that included biofeedback and intravaginal electrostimulation. Other evaluations included pelvic floor manometry, electromyography, and quality-of-life questionnaires, including the short forms of the Pelvic Organ Prolapse/Urinary Incontinence Sexual Questionnaire, Urogenital Distress Inventory, and Incontinence Impact Questionnaire.

**Outcomes:**

Clinical characteristics, vaginal squeezing and resting pressure, maximal pelvic floor contraction, duration of sustained contraction, quality-of-life scores, and sexual function were compared between baseline and after the pelvic floor muscle training programs.

**Results:**

There were 61 women included in the study. The mean number of treatment sessions was 12.9 ± 6.3, and the mean treatment duration was 66.7 ± 32.1 days. The short forms of the Urogenital Distress Inventory (7.7 ± 3.8 vs 1.8 ± 2.1; *P* < .001) and Incontinence Impact Questionnaire (5.9 ± 4.3 vs 1.8 ± 2.0; *P* < .001) scores significantly improved after the pelvic floor muscle training program. In addition, all pelvic floor muscle activities significantly improved, including maximal vaginal squeezing pressure (58.7 ± 20.1 cmH_2_O vs 66.0 ± 24.7 cmH_2_O; *P* = .022), difference in vaginal resting and maximal squeezing pressure (25.3 ± 14.6 cmH_2_O vs 35.5 ± 16.0 cmH_2_O; *P* < .001), maximal pelvic muscle voluntary contraction (24.9 ± 13.8 μV vs 44.5 ± 18.9 μV; *P* < .001), and duration of contraction (6.2 ± 5.7 s vs 24.9 ± 14.6 s; *P* < .001). Nevertheless, the short form of the Pelvic Organ Prolapse/Urinary Incontinence Sexual Questionnaire score demonstrated no significant improvement (28.8 ± 9.7 vs 29.2 ± 12.3; *P* = .752).

**Clinical Implications:**

Pelvic floor muscle training programs may not improve sexual function in women with stress urinary incontinence.

**Strengths and Limitations:**

The strength of this study is that we evaluated sexual function with validated questionnaires. The small sample size and lack of long-term data are the major limitations.

**Conclusion:**

Pelvic floor muscle training can improve pelvic floor muscle activities and effectively treat stress urinary incontinence; however, it may not improve sexual function.

## Introduction

Stress urinary incontinence (SUI) is defined as involuntary urine leakage on effort, exertion, sneezing, or coughing.^1^ SUI is a relatively common disorder and has negative impacts on quality of life. The pathophysiology of SUI is multifactorial, and one important factor is pelvic floor muscle weakness. Consequently, pelvic floor muscle training (PFMT), a conservative treatment, is recommended as first-line treatment.^2^ The principle of PFMT is to engage in the rehabilitation of the pelvic floor muscles, aiming to restore the functional integrity of the pelvic floor. However, even though PFMT is the most commonly used physical therapy for women with SUI, up to 30% of women may struggle to accurately contract pelvic floor muscles during the training.^3^ To overcome this problem, adjunctive therapies with biofeedback and/or electrostimulation are considered to be effective to strengthen the pelvic floor muscles in patients who are unable to contract properly and effectively.^3^

Besides playing a role in maintaining continence, pelvic floor muscles may also play an important role in sexual function, as they are responsible for the involuntary rhythmic contractions during orgasm.^4^ A few studies have reported that PMFT could improve not only incontinence-related quality of life, but also female sexual function.^5–7^ One randomized controlled trial evaluated women who had pelvic organ prolapse and underwent 6 months of PFMT, and the results showed that PFMT helped some of the women to increase control, strength, and awareness of their pelvic floor.^7^ Furthermore, the perception of a “tighter” vagina was associated with improved sexual desire and orgasms, and raised sexual gratification for their partners.^7^ Nevertheless, another randomized study failed to show an improvement in sexual function between patients who received a placebo and those who received several sessions of vaginal electrical stimulation.^4^ Therefore, the association between conservative management to strengthen pelvic floor muscles and female sexual function remains unclear. We hypothesized that PFMT would have a positive effect on pelvic floor muscle maximal contraction pressure, endurance of contraction, and vaginal squeezing pressure, and consequently that these improvements in muscle activities would lead to better incontinence-related quality of life, symptom severity, and sexual function. We aimed to address the following question: “What is the impact of PFMT programs on pelvic floor muscle activities and sexual function in women with SUI?”

## Methods

Patients who sought care at a urogynecologic outpatient clinic at a tertiary medical center and underwent PFMT programs from July 2014 to July 2015 were retrospectively reviewed by electronical medical record. All participants underwent urodynamic examination and 1-hour pad test. Sexual function was assessed by the short form of the Pelvic Organ Prolapse/Urinary Incontinence Sexual Questionnaire (PISQ-12).^8^ The PISQ-12 is a condition-specific questionnaire that evaluates sexual function in women who experience pelvic organ prolapse and/or urinary incontinence. There are 3 domains in PISQ-12: behavioral-emotive (items 1-4), physical (items 5-9), and partner-related (items 10-12). A total of 48 is the maximum score with higher scores indicate better sexual function. Additional assessments included detailed medical history, pelvic examination, ultrasound scan, and urine analysis. Pelvic examination was performed in lithotomic position, conducted by 2 urogynecologists in our hospital. Pelvic organ prolapse was described under maximal Valsalva according to pelvic organ prolapse quantification system and cough stress test was recorded as well. Incontinence-related symptom distress and quality of life were assessed using the short forms of the Urinary Distress Inventory (UDI-6) and Incontinence Impact Questionnaire (IIQ-7)^9^ on the 1st, 6th, 12th, 18th, and final sessions of treatment. The IIQ-7 scores provide the impact of urinary incontinence in quality of life and the UDI-6 score indicates overall symptom distress. Higher scores indicate greater symptom severity. We included patients who had urodynamically proven SUI, those who were willing to undergo a program of PFMT with biofeedback and electrical stimulation, and those who completed all evaluations. Exclusion criteria comprised individuals with mixed incontinence, those with pelvic organ prolapse stage II or above, those with an ongoing vaginal or urinary tract infection, and those who were unable to contract their pelvic floor muscles due to a neurological disorder or cognitive deficit. This study was approved by the Institutional Review Board of our hospital (14MMHIS031).

The PFMT programs included biofeedback and electrostimulation, which were carried out 2 times per week, with each course comprising 6 sessions. Before the training program, patients received an explanation of pelvic floor anatomy and were instructed on the correct technique for contracting pelvic floor muscles by a physiotherapist. Each session began with the measurement of baseline vaginal squeezing pressure using a manometer (Medical Measurement System). Electromyography was recorded and analyzed using Wireless Patient Module (Medical Measurement System). Two surface electrodes were positioned at 3 and 9 o’clock laterally from the anal sphincter, forming one channel, while another channel connected 2 surface electrodes were placed laterally to the umbilicus. Pelvic floor muscle strength involved assessing maximum voluntary contraction, maximum duration of sustained contraction, and synergic abdominal muscles, which were measured by electromyography during each contraction. The biofeedback protocol consists of 2 phases: (1) fast contraction lasting for 1 second, followed by 2 seconds rest, repeated 15 to 20 times depending on the patient’s tolerance; and (2) a 1-minute rest, followed by sustained contraction lasting for 5 seconds, with a subsequent 10-second rest, repeated 15 to 20 times. The duration of sustained contractions varied based on patients’ tolerance. Electrical stimulation was performed by FemiScan Stim with a Periform vaginal electrode probe (Danmeter) and followed the biofeedback. The stimulation parameters were set at a frequency of 35 Hz, pulse width of 250 μS, stimulation time of 5 seconds, and interval time of 10 seconds. The output current was adjusted according to the patients’ maximal tolerable intensity (maximum 100 mA), and the electrical stimulation treatment last for 20 minutes. After electrical stimulation, patients were educated on using an indicator stick connected to a vaginal probe to provide visual feedback when the muscles were contracting correctly. Patients were instructed to practice minimum of 3 sets of 10 to 15 repetitions of pelvic floor muscle exercise per day at home.

Comparisons were made between baseline and the 6th, 12th, 18th, and final treatment sessions for changes in UDI-6, IIQ-7, and PISQ-12 scores. Additionally, vaginal squeezing pressure, pelvic floor muscle maximal voluntary contraction pressure, and duration of sustained contractions were analyzed. Statistical analysis was conducted using the independent *t* test to assess the means of continuous variables, while the independent *t* test or Mann-Whitney *U* test was utilized for continuous variables. A *P* value <.05 was considered to be statistically significant. The Statistical analysis was conducted using SPSS version 20 (IBM).

## Results

There were 61 women included in the study. The mean number of treatment sessions was 12.9 ± 6.3, and the mean treatment duration was 66.7 ± 32.1 days. At least 6 sessions of PFMT with biofeedback and electrostimulation were completed for each patient. The demographic characteristics and baseline conditions are shown in Table 1. Changes in incontinence-related quality of life, symptom distress, electromyographic activities, vaginal squeezing pressure, and sexual function before and after the last session of PFMT are shown in Table 2. For the incontinence-related quality of life and symptom distress, the UDI-6 (7.7 ± 3.8 vs 1.8 ± 2.1; *P* < .001) and IIQ-7 (5.9 ± 4.3 vs 1.8 ± 2.0; *P* < .001) scores significantly improved after the PFMT program. However, there was no significant improvement in PISQ-12 score (28.8 ± 9.7 vs 29.2 ± 12.3; *P* = .752). All pelvic floor muscle measurements significantly improved after the PFMT program, including maximal vaginal squeezing pressure (58.7 ± 20.1 cmH_2_O vs 66.0 ± 24.7 cmH_2_O; *P* = .022), difference in vaginal resting and maximal squeezing pressure (25.3 ± 14.6 cmH_2_O vs 35.5 ± 16.0 cmH_2_O; *P* < .001), maximal pelvic muscle voluntary contraction (24.9 ± 13.8 μV vs 44.5 ± 18.9 μV; *P* < .001), and duration of contraction (6.2 ± 5.7 s vs 24.9 ± 14.6 s; *P* < .001). However, although vaginal resting pressure improved, this difference was not statistically significant (33.3 ± 12.2 vs 36.0 ± 15.0; *P* = .061). Our primary outcome was the UDI-6 score, and patients who had improvement in stress incontinence discontinued the PFMT program. The dropout rate was 23.0 %.

**Table 1:** Characteristics of women undergoing the pelvic floor muscle training with biofeedback and electrostimulation (N = 61).

Age, y	50.9 ± 11.7
Parity	2.2 ± 1.1
Vaginal delivery	17 (77)
Cesarean section	4 (18)
Body mass index, kg/m^2^	23.6 ± 3.8
Hypertension	4 (18)
Diabetes	2 (9)
Postmenopausal status	10 (45)
Hormonal therapy	2 (9)
Quality of life	
PISQ-12	28.8 ± 9.7
UDI-6	7.7 ± 3.8
IIQ-7	5.9 ± 4.3
Number of treatment sessions	12.9 ± 6.3
Total duration of treatment, d	66.7 ± 32.1
Pelvic muscle floor electrophysiological condition	
Vaginal resting pressure, cmH_2_O	33.3 ± 12.2
Vaginal squeezing pressure, cmH_2_O	58.7 ± 20.1
Difference of vaginal resting and squeezing pressure, cmH_2_O	25.3 ± 14.6
Maximal voluntary contraction, μV	24.9 ± 13.8
Duration of contraction, s	6.2 ± 5.7

Values are mean ± SD or n (%).

Abbreviations: IIQ-7, short form of the Incontinence Impact Questionnaire; PISQ-12, short form of the Prolapse/Urinary Incontinence Sexual Questionnaire; SUI, stress urinary incontinence; UDI-6, short form of the Urogenital Distress Inventory.

**Table 2:** The change of quality of life and pelvic electrophysiological condition before and after the last session of biofeedback and electrostimulation.

	Before treatment (n = 61)	After treatment (n = 47)	*P*
PISQ-12	28.8 ± 9.7	29.2 ± 12.3	.752
UDI-6	7.7 ± 3.8	1.8 ± 2.1	<.001
IIQ-7	5.9 ± 4.3	1.8 ± 2.0	<.001
Vaginal resting pressure, cmH_2_O	33.3 ± 12.2	36.0 ± 15.0	.061
Vaginal squeezing pressure, cmH_2_O	58.7 ± 20.1	66.0 ± 24.7	.022
Difference of vaginal resting and squeezing pressure, cmH_2_O	25.3 ± 14.6	35.5 ± 16.0	<.001
Maximal voluntary contraction, μV	24.9 ± 13.8	44.5 ± 18.9	<.001
Duration of contraction, s	6.2 ± 5.7	24.9 ± 14.6	<.001

Values are mean ± SD.

Abbreviations: IIQ-7, short form of the Incontinence Impact Questionnaire. PISQ-12, short form of the Prolapse/Urinary Incontinence Sexual Questionnaire; UDI-6, short form of the Urogenital Distress Inventory.

Table 3 presents the comparisons between the baseline and 6th, 12th, and 18th or more sessions. Notably, there were significant reductions in UDI-6 (7.3 ± 3.1 vs 5.1 ± 3.5, 3.7 ± 2.7, and 2.8 ± 2.6; *P* = .009, <.001, and .001, respectively) and IIQ-7 (8.3 ± 5.8 vs 4.8 ± 5.2, 3.9 ± 4.0, and 2.4 ± 2.1; *P* = .002, <.001, and .001, respectively) scores following each set of 6 treatment sessions. However, there was no significant improvement in the PISQ-12 score from baseline regardless of the number of treatment sessions (28.8 ± 9.7 vs 30.4 ± 10.1, 30.2 ± 11.7, and 26.9 ± 7.9; *P* = .357, .434, and .236, respectively). While UDI-6 and IIQ-7 scores significantly decreased, all pelvic floor muscle measurements significantly improved, including vaginal squeezing pressure (58.7 ± 20.1 cmH_2_O vs 67.4 ± 23.7 cmH_2_O, 79.1 ± 26.5 cmH_2_O, and 63.7 ± 24.6 cmH_2_O; *P* = .013, <.001, and .037, respectively), difference in vaginal resting and squeezing pressure (25.3 ± 14.6 cmH_2_O vs 31.1 ± 14.5 cmH_2_O, 40.5 ± 20.9 cmH_2_O, and 31.6 ± 17.4 cmH_2_O; *P* = .022, <.001, and .019, respectively), maximal voluntary contraction (29.2 ± 17.6 μV vs 35.2 ± 27.7 μV, 39.9 ± 26.3 μV, and 45.2 ± 29.2 μV; *P* = .006, .003, and <.001, respectively), and duration of sustained contraction (6.2 ± 5.7 s vs 8.7 ± 7.0 s, 16.3 ± 11.7 s, 39.9 ± 27.9 s; *P* = .037, <.001, and <.001, respectively). However, no significant difference was noted in vaginal resting pressure (33.3 ± 12.2 cmH_2_O vs 36.7 ± 14.0 cmH_2_O, 38.6 ± 13.3 cmH_2_O, and 32.1 ± 13.8 cmH_2_O; *P* = .067, .041, and .728, respectively).

**Table 3:** Comparison of electromyography measurements and quality of life before and after treatment sessions.

	1st (Baseline)	6th	12th or more	18th or more	*P*		
					6th vs baseline	12th vs baseline	18th vs baseline
Number of patients	61	39	22	19			
Vaginal resting pressure, cmH_2_O	33.3 ± 12.2 (2, 70)	36.7 ± 14.0 (2, 62)	38.6 ± 13.3 (2, 58)	32.1 ± 13.8 (1, 62)	.067	.041	.728
Vaginal squeezing pressure, cmH_2_O	58.7 ± 20.1 (7, 115)	67.4 ± 23.7 (8, 132)	79.1 ± 26.5 (6, 132)	63.7 ± 24.6 (15, 125)	.013	<.001	.037
Difference of vaginal resting and squeezing pressure, cmH_2_O	25.3 ± 14.6 (1, 60)	31.1 ± 14.5 (6, 70)	40.5 ± 20.9 (9, 74)	31.6 ± 17.4 (13, 86)	.022	<.001	.019
Maximal voluntary contraction, μV	29.2 ± 17.6 (3, 92)	35.2 ± 27.7 (3, 140)	39.9 ± 26.3 (6, 138)	45.2 ± 29.2 (6, 132)	.006	.003	<.001
Duration of sustained contraction, s	6.2 ± 5.7 (1.3, 38.3)	8.7 ± 7.0 (2.4, 43.3)	16.3 ± 11.7 (5.3, 72.6)	39.9 ± 27.9 (5.9, 119.2)	.037	<.001	<.001
PISQ-12	28.8 ± 9.7 (0, 42)	30.4 ± 10.1 (2, 44)	30.2 ± 11.7 (2, 44)	26.9 ± 7.9 (4, 36)	.357	.434	.236
UDI-6	7.3 ± 3.1 (0, 30)	5.1 ± 3.5 (0, 12)	3.7 ± 2.7 (0, 14)	2.8 ± 2.6 (0, 16)	.009	<.001	<.001
IIQ-7	8.3 ± 5.8 (0, 29)	4.8 ± 5.2 (0, 18)	3.9 ± 4.0 (0, 18)	2.4 ± 2.1 (0, 20)	.002	<.001	<.001

Values are n or mean ± SD (minimum, maximum).

Abbreviations: IIQ-7, short form of the Incontinence Impact Questionnaire. PISQ-12, short form of the Prolapse/Urinary Incontinence Sexual Questionnaire; UDI-6, short form of the Urogenital Distress Inventory.

We calculate power analysis to define our sample size. As for the treatment of stress urinary incontinence, our primary outcome is the UDI-6. According to the clinical experience, we assumed that the scores of mean ± SD in SUI before and after treatment are 5.0 ± 2.0 and 4.15 ± 2.0 in UDI-6, respectively. The effect size we calculated was 0.425, and to achieve a power of >80% with an α value <0.05 (2-sided), a sample size of 46 was adequate for the powered study by using software G*Power version 3.1 (Franz Faul University).

## Discussion

In this study, we found that PFMT significantly improved pelvic floor muscle strength, alleviated distress associated with incontinence symptoms, and positively impacted quality of life. However, patients’ sexual function was not significantly influenced by PFMT.

Several studies have reported that PFMT can increase sexual satisfaction.^10–13^ Bø et al^11^ evaluated 59 women with clinically and urodynamically proven stress incontinence randomized to either a 6-month PFMT or control group, and found statistically significant reductions in the number of women having problems with their sex life, social life, and physical activity in the PFMT group. Echoing these results, a retrospective study conducted by Lowenstein et al^12^ investigated 176 women with a primary complaint of sexual dysfunction and found that orgasm and arousal function were related to better pelvic floor muscle function. Similar findings were reported by another cross-sectional study by Martinez et al,^13^ who investigated the relationship between pelvic floor muscle strength and sexual function among 40 women and found that women with stronger pelvic floor muscles had better sexual function. However, Bø et al^11^ investigated sexual function as a secondary outcome, and there were no baseline data to compare the difference in sexual function before and after treatment. In addition, the mean ages in the other 2 cohort studies were relatively younger than average urogynecologic patients,^12^^,^^13^ at 37 ± 11 and 23.2 ± 3.2 years, respectively. Therefore, these results may not be generalizable to patients with pelvic floor dysfunction, such as those with incontinence and/or pelvic organ prolapse. A systematic review consequently suggested that the efficacy of PFMT to improve female sexual function should be interpreted with caution.^10^

To date, there is no consensus in the literature to explain the mechanism of how PFMT improves female sexual function,^10^ and the results of previous studies have been conflicting.^14–16^ A randomized control trial conducted by Wilson and Herbison^14^ recruited 230 women who were incontinent and randomized them into a control group and an intervention group, which included PFMT, vaginal cones, or combined therapy. Consistent with our findings, pelvic floor muscle contraction strength was significantly improved at 1 year of follow-up. However, no notable difference was observed in sexual satisfaction, encompassing aspects such as dyspareunia, satisfaction, interest in sex, arousal, and the ability to achieve orgasm between the 2 groups.^14^ Another study reported that pelvic floor muscle exercises did not assist sexual performance.^15^ In that study, Roughan and Kunst^15^ recruited 46 women allocated to 1 of 3 groups, namely a pelvic floor exercise, relaxation, or control group. Pubococcygeal muscle tone was measured and questionnaires about sexual response were completed at 12 weeks and 6 months of follow-up, and the results showed that there were no significant differences in orgasmic outcome between the groups during the experimental period. Another study exploring the association of baseline pelvic floor muscle activities with urinary and sexual functions in women with SUI may explain the results.^16^ Yang et al^16^ prospectively reviewed 125 sexually active women with SUI who underwent intravaginal digital examinations and pelvic ultrasound to detect pelvic floor activities, and their responses to questionnaire surveys including the UDI-6, IIQ-7, and PISQ-12. They found that higher pelvic floor muscle contraction strength was not associated with better sexual or urinary function. Similarly, we found independent results between pelvic muscle activities and female sexual function.

The results of the current study showed that PFMT is a promising management approach for incontinence because the incontinence-related quality of life and symptom distress significantly improved, and with a dose-dependent effect. Regular and targeted exercise could strengthen pelvic floor muscle activities and effectively rebuild function. Therefore, we suggest that PFMT could be used as the first-line treatment for urinary incontinence. For sexual function, however, we suggest that a larger randomized control trial should be carried out before PFMT is employed for this purpose. Because female sexuality is very complex and can be influenced by biological, sociocultural, and partner- and psychosexual-related factors, further studies are need to clarify our results.

The strength of this study is that we excluded patients with pelvic organ prolapse, which made our study population more homogeneous. We also used valid questionnaires to evaluate the outcomes. The PFMT program was carried out by physiotherapist with same protocol, reducing the bias within supervised training. There are some limitations include small sample size and lack of a control group. This study is also limited by lacking baseline sexual characteristics, as sexual function is also influenced by sexual activity and intimacy. Nevertheless, patients who answered the PISQ-12 were sexually active over the past 6 months.

## Conclusion

PFMT can improve pelvic floor muscle activities and effectively manage SUI. However, PFMT may not improve sexual function in women with SUI.
